# Supplementary material for: High throughput variant libraries and machine learning yield design rules for retron gene editors
Source: Nucleic Acids Res. 2024 Dec 10;53(2):gkae1199. doi: 10.1093/nar/gkae1199 (PMC11754653; doi:10.1093/nar/gkae1199)
Supplement: gkae1199_Supplemental_Files [file gkae1199_supplemental_files.zip › REVISION_SUPPLEMENTAL_NAR_Variant_and_ML_Manuscript.pdf]

Supplementary Figures

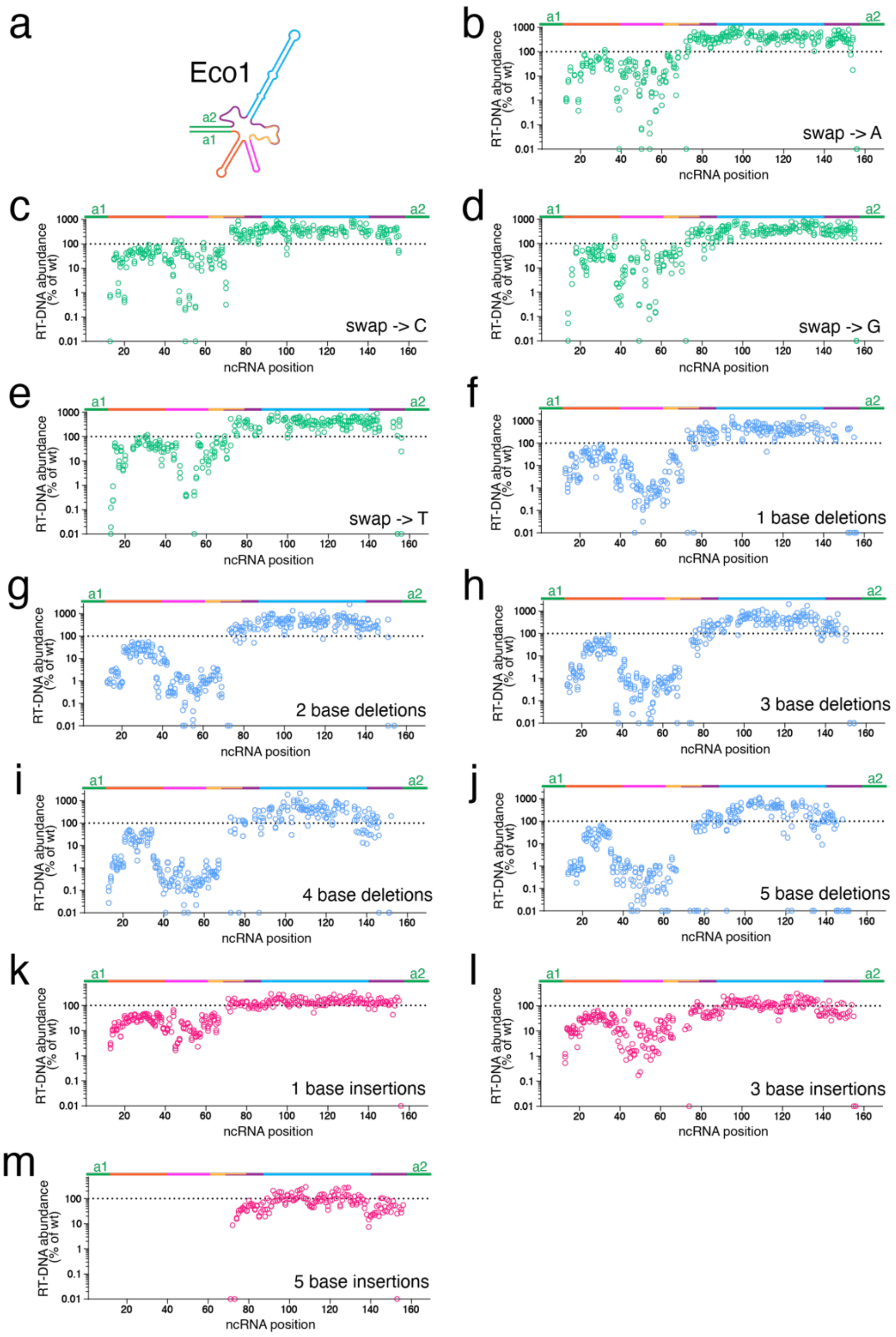

Supplementary Figure 1. Substitution, deletion, and insertion sub-library msDNA production in *E. coli*. a. Retron-Eco1 ncRNA structure. **b.** msDNA production of N→A nucleotide swap, starting at a specified ncRNA position relative to wild-type msDNA. Each open circle represents an individual biological replicate. **c.** msDNA production of N→C nucleotide swap, starting at a specified ncRNA position relative to wild-type msDNA. Each open circle represents an individual biological replicate. **d.** msDNA production of N→G nucleotide swap, starting at a specified ncRNA position relative to wild-type msDNA. Each open circle represents an individual biological replicate. **e.** msDNA production of N→T nucleotide swap, starting at a

specified ncRNA position relative to wild-type msDNA. Each open circle represents an individual biological replicate. **f.** msDNA production of single-base deletions, starting at a specified ncRNA position relative to wild-type msDNA. Each open circle represents an individual biological replicate. **g.** msDNA production of two-base deletions, starting at a specified ncRNA position relative to wild-type msDNA. Each open circle represents an individual biological replicate. **h.** msDNA production of 3-base deletions, starting at a specified ncRNA position relative to wild-type msDNA. Each open circle represents an individual biological replicate. **i.** msDNA production of 4-base deletions, starting at a specified ncRNA position relative to wild-type msDNA. Each open circle represents an individual biological replicate. **j.** msDNA production of 5-base deletions, starting at a specified ncRNA position relative to wild-type msDNA. Each open circle represents an individual biological replicate. **k.** msDNA production of single-base insertions, starting at a specified ncRNA position relative to wild-type msDNA. Each open circle represents an individual biological replicate. **l.** msDNA production of 3-base insertions, starting at a specified ncRNA position relative to wild-type msDNA. Each open circle represents an individual biological replicate. **m.** msDNA production of 5-base insertions, starting at a specified ncRNA position relative to wild-type msDNA. Each open circle represents an individual biological replicate.

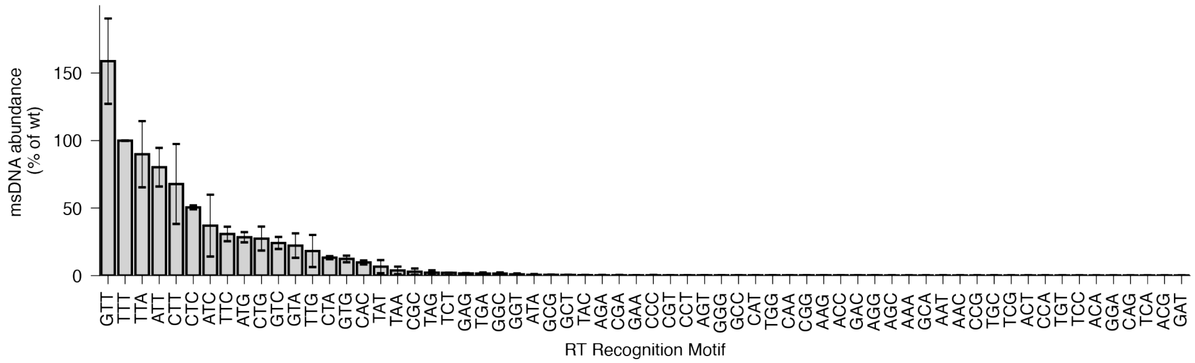

Supplementary Figure 2. msDNA production of every permutation of Retron-Eco1 reverse transcriptase recognition motif as a barplot. msDNA production is relative to wild-type msDNA abundance. Each bar represents the mean of three biological replicates. There is a significant effect of the RT recognition motif (one-way ANOVA, P<0.0001), with every permutation significantly different than the wild-type UUU (P<0.0001) except UUA and AUU (P=0.8991 and P=0.0551, respectively) (Dunnett's, corrected). Data is the same as is presented in **Fig 1k**.

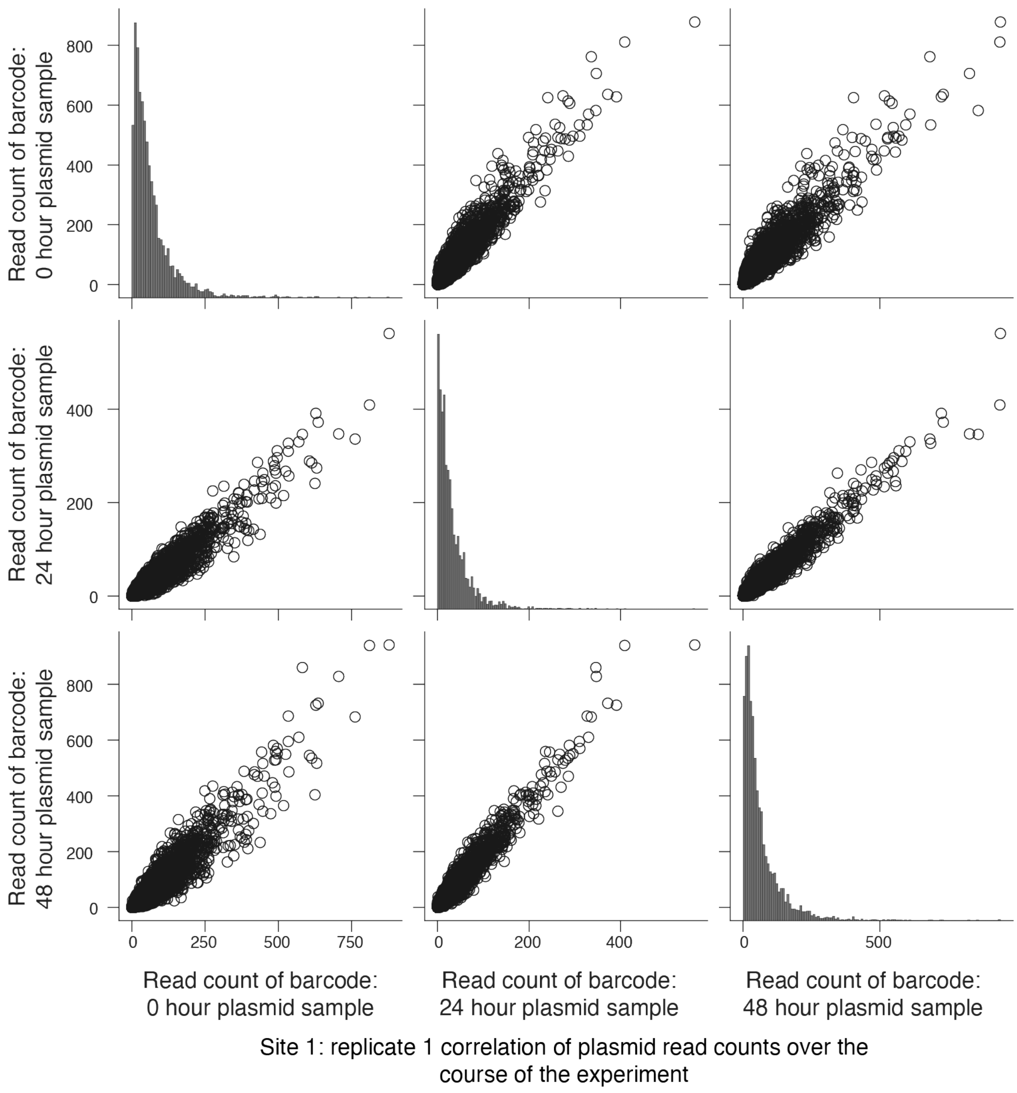

Supplementary Figure 3. Correlation in plasmid read counts over an example 48-hr editing window in *S. cerevisiae*. Correlation between individual plasmid barcode read counts at 0 hr, 24 hr, and 48 hr of editing for the first biological replicate of the site 1 library. Each open circle represents an individual barcode read count.

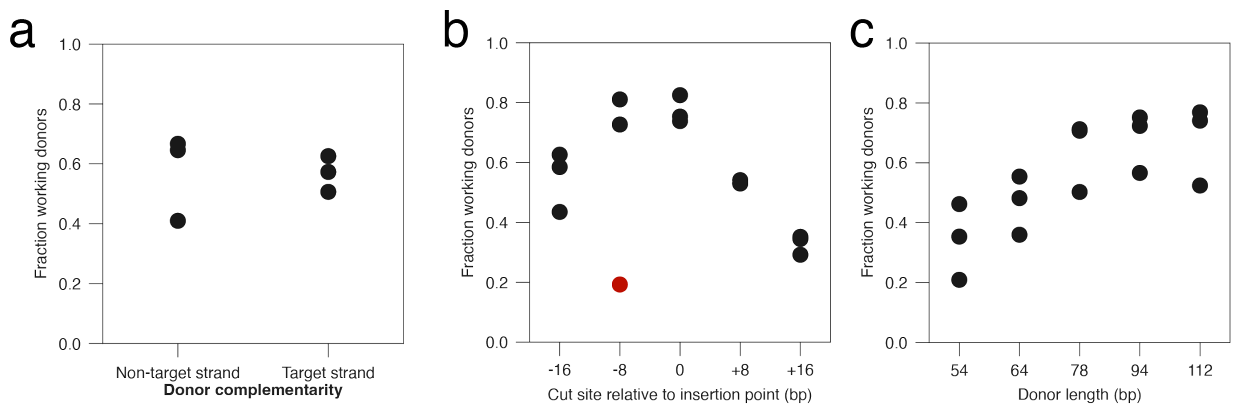

Supplementary Figure 4. Fraction working editors for different editing variables. a. Fraction working donors that are complementary to the non-target strand vs. target strand when reverse-transcribed into donor DNA. Each closed circle represents the mean of the three biological replicates for that site. **b.** Fraction working donors across all tested cut sites. Each closed circle represents the mean of the three biological replicates for that site. The red closed circle represents the cut site excluded from the analysis, as the fraction working donors is below 20%. **c.** Fraction working donors across all tested donor lengths. Each closed circle represents the mean of the three biological replicates for that site.

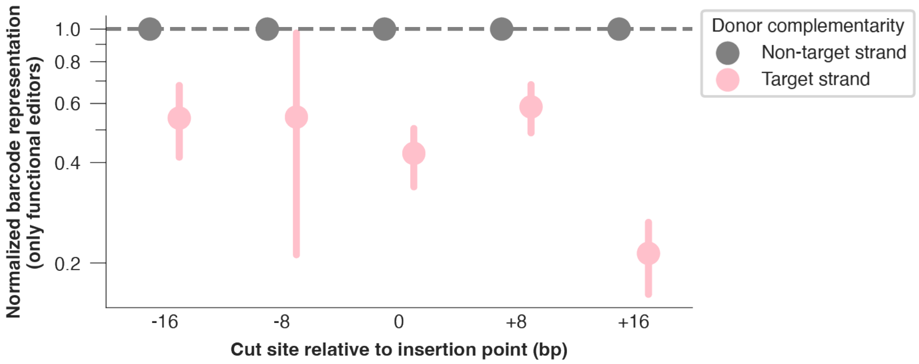

Supplementary Figure 5. Normalized barcode representation of target and non-target strand donors broken out by cut site. All target-strand-homologous gRNA/donor variants' barcode representation normalized against its non-target strand homologous gRNA/donor variant for every cut site (±standard deviation), with all other variables held constant (chassis, donor length, and center). The variants for each site are plotted in different colors, and each biological replicate of a site is summarized by the median (left) of the distribution of variants (right). Data is the same as presented in **Fig 3e.**

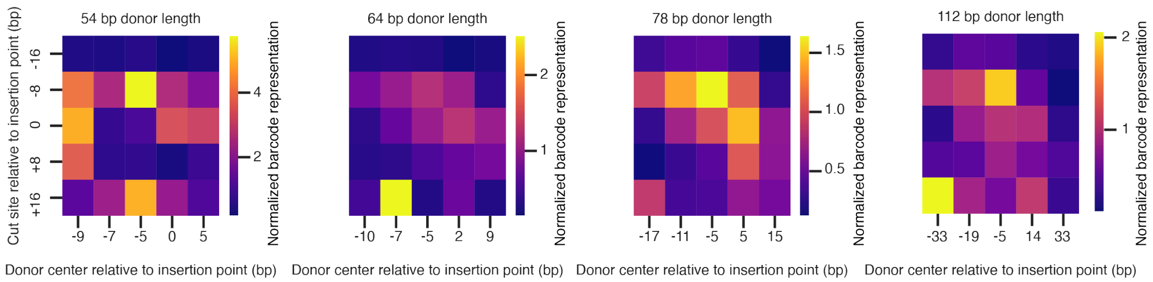

Supplementary Figure 6. Normalized barcode representation of donors of varying cut sites vs. donor centers in *S. cerevisiae*. Heat map of normalized barcode representation of cut site vs. donor center (54, 64, 78, and 112 nucleotide donor length), normalized to the cut site at the barcode insertion site, and donor center of -5 bp from the barcode insertion site. Each square represents the mean of all biological replicates across all sites.

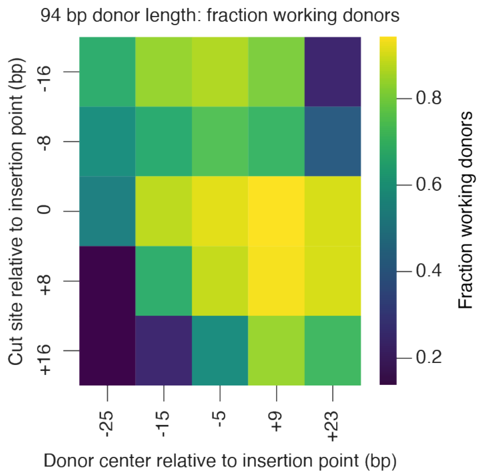

Supplementary Figure 7. Standard deviation of normalized barcode representation of donors of varying cut sites vs. donor centers in *S. cerevisiae*. Heat map of the standard deviation of normalized barcode representation of cut site vs. donor center (94 nucleotide donor length), normalized to the cut site at the barcode insertion site, and donor center of -5 bp from the barcode insertion site. Each square represents the standard deviation of all biological replicates across all sites.

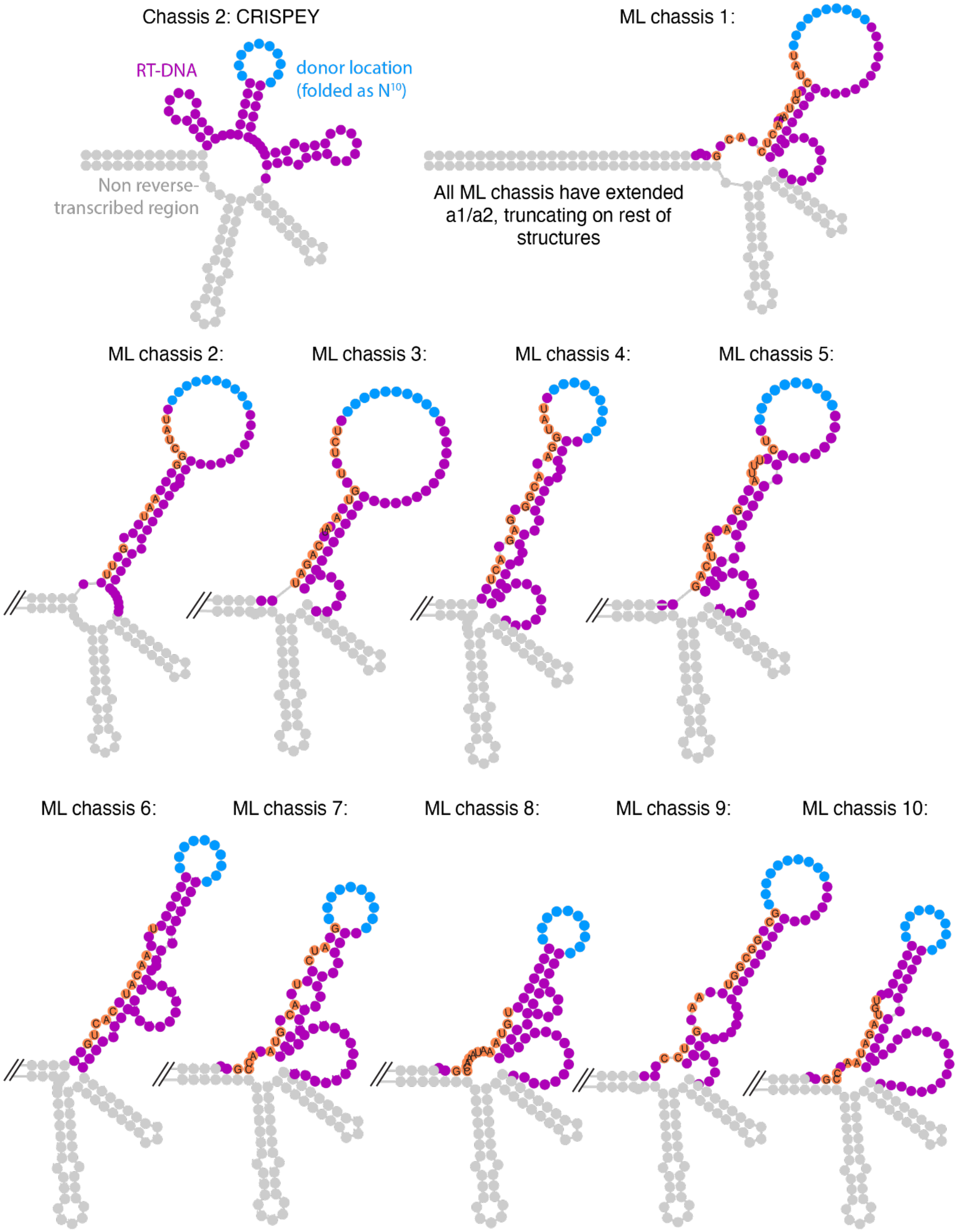

Supplementary Figure 8. ncRNA structure and sequence of top machine learning ncRNA chassis. CRISPEY and ML chassis were folded using RNAfold (Institute for Theoretical Chemistry, University of Vienna webtool) using N_{10} to stand in for the variable donor region (light blue). *msr* annotated in grey and

msDNA annotated in purple. Nucleotides with changes from the CRISPEY reference are highlight in red with the nucleotide identity annotated in black.

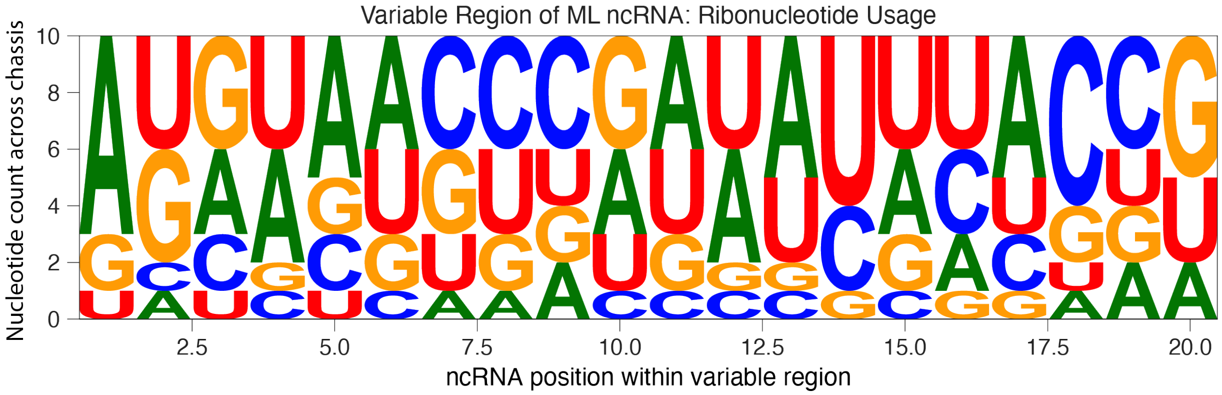

Supplementary Figure 9. Usage of ribonucleotides in ML ncRNA chassis across variable region. Ribonucleotide height scaled with usage, created by the Python logomaker package.

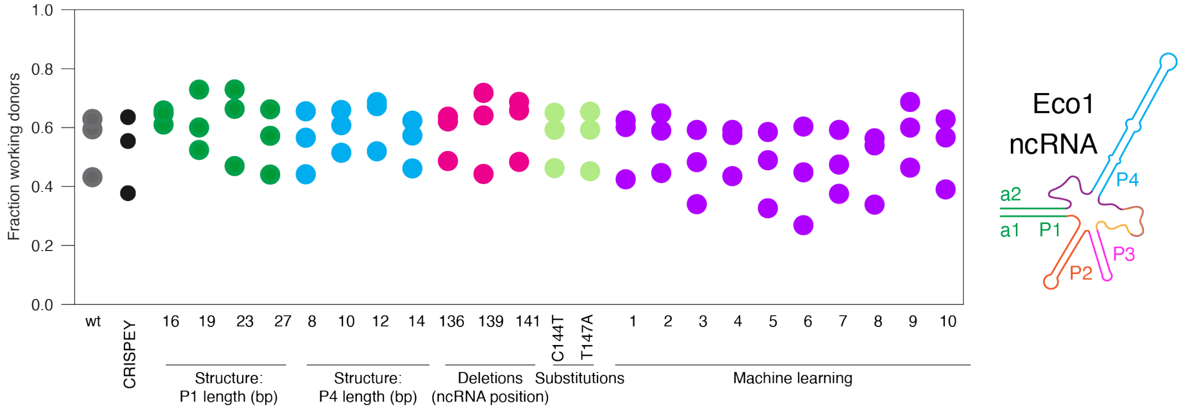

Supplementary Figure 10. Fraction working editors across ncRNA chassis. Fraction of working donors across all ncRNA chassis. Each closed circle represents the mean of the three biological replicates for that site.

Supplementary Table Legends:

Supplementary_Tables _1-5.xlsx

This PDF file contains:

- Supplementary Table 1: Statistical analysis
- Supplementary Table 2: Plasmids used in this study
- Supplementary Table 3: Strains used in this study
- Supplementary Table 4: Primers used in this study
- Supplementary Table 5: Donors used in this study

Supplementary_Tables _6-8.xlsx

- Supplementary Table 6: Donors used for Site 1 *S. cerevisiae* library
- Supplementary Table 7: Donors used for Site 2 *S. cerevisiae* library
- Supplementary Table 8: Donors used for Site 3 *S. cerevisiae* library

Supplementary_Tables _9-18.xlsx

- Supplementary Table 9: ncRNAs used for **Fig 1c,f** and **Extended Data Fig 1b-e** (*msd*)
- Supplementary Table 10: ncRNAs used for **Fig 1c,f** and **Extended Data Fig 1b-e** (*msr*)
- Supplementary Table 11: ncRNAs used for **Fig 1d,f** and **Extended Data Fig 1f-j** (*msd*)
- Supplementary Table 12: ncRNAs used for **Fig 1d,f** and **Extended Data Fig 1f-j** (*msr*)
- Supplementary Table 13: ncRNAs used for **Fig 1e,f** and **Extended Data Fig 1k-m** (*msd*)

- Supplementary Table 14: ncRNAs used for **Fig 1e,f** and **Extended Data Fig 1k-m** (*msr*)
- Supplementary Table 15: ncRNAs used for **Fig 1g**
- Supplementary Table 16: ncRNAs used for **Fig 1h**
- Supplementary Table 17: ncRNAs used for **Fig 1i**
- Supplementary Table 18: ncRNAs used for **Fig 1k**
